# Supplementary material for: Direct medical costs after surgical or nonsurgical treatment for degenerative lumbar spinal disease: A nationwide matched cohort study with a 10-year follow-up
Source: PLoS One. 2021 Dec 1;16(12):e0260460. doi: 10.1371/journal.pone.0260460 (PMC8635587; doi:10.1371/journal.pone.0260460)
Supplement: Attachment — Submitted filename: Response to Reviewers 20210804.docx [file pone.0260460.s002.docx]

Authors’ revision letter

Please use the space provided to explain your answers to the questions above. You may also include additional comments for the author, including concerns about dual publication, research ethics, or publication ethics. (Please upload your review as an attachment if it exceeds 20,000 characters)

Reviewer #1:

1. This study reviews the cost of surgical versus non-surgical intervention on lumbar degenerative spine pathology in a national database. The authors utilize Korea’s national health insurance data repository to evaluate fee for service charges. The authors find that, while surgery is initially more expensive, the results even out over years.
   The manuscript offers good analysis of a national insurance database. It is certainly novel, and the results likely warrant publication. However, there are some issues.

Answer: I appreciate these encouraging comments. Please see our changes throughout the revised manuscript.

2. When matching patients, the authors use age, sex, diagnosis and the presence of osteoporosis and diabetes as variables. The authors don’t give a rationale for these comorbidities. They leave out several important comorbidities (for example, smoking status, hypertension, CHF, etc).

Answer: I appreciate this helpful comment. As you mentioned, other factors are also correlated with the outcomes, and those factors should have been considered in the matching process. The purpose of this study was to provide an overview of the utilization of the National Health Insurance System for degenerative lumbar spinal disease by showing the direct medical cost of surgery and nonsurgical treatment. In this regard, the data set for the analysis was to include patients with spinal pathology as a primary or secondary diagnosis and without previous surgery. We tried to match two groups (surgery and nonsurgical treatment) by using available factors that were associated with disease severity and outcomes. Many diagnoses are registered in the repository, but diagnoses directly linked to each direct cost are registered as a primary or secondary diagnosis in a customary fashion. All other comorbidities could not be tagged along with data set. Because comorbidities listed in Charlson’s comorbidity index appeared in more than 50% of patients due to fees for the service system of the NHIS, including all listed comorbidities was not practical in an analysis of secondary data of the NHIS. Unfortunately, smoking status is not registered in the data repository. We specifically included osteoporosis and diabetes because those factors were correlated with outcomes in our previous analyses (Kim et al. Spine 2013; Kim et al. Spine J 2013; Kim et al. Spine 2015; Kim et al. World Neurosurg 2018, Kim et al. Spine, 2019) Nonetheless, those factors should have been included in the matching process, or a rationale for limiting matching factors to the ones we used should have been explained. Unfortunately, access to the repository was allowed for a limited period, and it will take another year to obtain research funding and reapproval from the NHIS for additional analysis. Nonetheless, we think that the current result would be sufficient to show an overview regarding the utilization of resources. However, we acknowledge the limitation of using secondary data and clearly note that the purpose of this study is to provide an overview of the two groups. Consequently, the results should not be directly applied in clinical practice or policy making, and we toned down the meaning of this study. We have clarified these issues in the manuscript and describe them in the discussion and limitation sections below.

Page 6, line 17

The objective of the present study was to ~~evaluate~~ provide an overview of the utilization of the NHIS for degenerative lumbar spinal disease by ~~comparing~~ reporting the direct medical costs of patients who underwent surgery and of patients who did not undergo surgery.

Page 8, line 21

disease codes as a primary or secondary diagnosis (LDH, M51, M472; lumbar spinal stenosis without spondylolisthesis, M4800, M4805-8; lumbar spinal stenosis with spondylolisthesis, M431, M4315-9; spondylolysis, M430, M4306-9),

Page 18, line 4

The objective of the present study was to ~~compare~~ provide an overview of the medical costs after treatment for degenerative lumbar conditions of the surgery and nonsurgical treatment cohorts.

Page 18, line 9

The results of the present study need to be interpreted with consideration of the cost of medical services, indirect costs, societal cost such as missed work or patient quality of life, societal willingness to pay (WTP), the guidelines of insurance institutes, and the limitations of the analysis in terms of its use of secondary data, and these issues will be discussed [20].

Page 21, line 16

Fourth, only age, sex, diagnosis, osteoporosis, diabetes and previous direct medical costs were considered for the matching process, and other important comorbidities, such as smoking, hypertension and congestive heart failure, and socioeconomic factors such as income and employment status, were not considered. Although the result seemed to provide a general overview of the direct medical costs, the current results posed a risk of bias due to potential incomplete matching. Regardless of these limitations, the present study provides an overview of how the NHIS is utilized for lumbar degenerative spinal disease on a nationwide scale, and this information may be helpful for physicians, patients and policy makers to improve the NHIS.

3. Furthermore, while it may not be available in their database, some sort of socioeconomic matching variable would be beneficial (insurance carrier, income, employment status, etc.).

Answer: I appreciate this valuable comment. Socioeconomic factors are also important factors that determine a direct medical costs. Fortunately, all Korean people are beneficiaries of the national health insurance system (NHIS), and access to medical services is not limited by insurance carriers. There are many nonsurgical treatments that are not covered by the NHIS, but noncovered costs were not considered in this paper. Unfortunately, other factors, such as income and employment status, also influence both direct and indirect medical costs, but those data were not registered in the repository and could not be analyzed. We clarified this in the limitations section.

Page 21, line 16

Fourth, only age, sex, diagnosis, osteoporosis, diabetes and previous direct medical costs were considered for the matching process, and other important comorbidities, such as smoking, hypertension and congestive heart failure, and socioeconomic factors such as income and employment status, were not considered. Although the result seemed to provide a general overview of the direct medical costs, the current results posed a risk of bias due to potential incomplete matching.

4. At the very least, further explanation of their process for arriving at the matching variables is needed. When matching the patients, are the costs over the last three years just regarding their spinal pathology or all medical costs?

Answer: Thank you for this question. We described the matching process in the revised manuscript. The cost implied a direct medical cost incurred during the previous 3 years with a primary or secondary diagnosis of spinal degenerative disease. Please see the changes as follows.

Page 6, line 23

A matched cohort study design was used for the present study. Initially, patients were selected from the NHIS-NSC on the basis of diagnosis and surgery codes. Thereafter, the following factors were considered for matching: age, sex, diagnosis, osteoporosis without fractures and diabetes mellitus, and the total cost incurred over the previous 3 years (which was assumed to be a surrogate for disease severity) [9, 10, 13, 14]. Osteoporosis and diabetes mellitus were specifically included for matching, because those factors were associated with outcomes in previous studies [9, 10, 13, 14]. Because clam data included many disease codes without information of severity, the other codes were not included in matching process. To match with limited factors, the categorical factors were matched exactly. The continuous variables were matched with a difference of no more than 3 years for age and 10% of standardized difference of total direct medical cost incurred in patients with surgery over the previous 3 years by lumbar spinal degenerative disease. The matching within 10% of the standard deviation of total direct medical cost was to make the standardized difference between the two groups less than 10%, which is considered well balanced between two groups.

Page 9, line 4

During the last 3 years, the standard deviation of the direct medical cost in 3,881 patients was $730, and a difference of no more than $73 was considered a similar severity of disease in the matching process.

5. The description of the “non-surgery” cohort as the “intervention” cohort is confusing. Why did the authors choose this label instead of something like “nonsurgical” or “conservative management?”

Answer: I apologize for mixing up these labels. The nonsurgical cohort and nonsurgical management (treatment) are more natural than nonsurgery, and I consistently use the nonsurgical label throughout the revised manuscript. Thank you.

6. The authors do mention this, but it is worth reiterating: the costs here do not account for societal costs such as missed work and quality of life.

Answer: Your comment is very important for interpreting the current result. We appreciate it, have reiterated this in the manuscript and have clarified that the cost used in this manuscript is the direct cost.

Page 9, line 15

Data on the direct medical cost used for both Western and Oriental medicine were retrieved from the database. The costs here do not account for societal costs such as missed work or quality of life.

Page 15, line 1

The average monthly direct medical cost in the surgery cohort was $50.84/patient/month, while the direct medical cost in the nonsurgical cohort was $29.34/patient/month (p < 0.01) (Table 3).

Page 21, line 4

Common clinical indicators such as quality of life or disability index would be helpful to better compare surgery and nonsurgical management.

Page 21, line 10

Third, the costs here do not account for uninsured costs or societal costs such as missed work or quality of life. [13, 48].

7. Lastly, the authors use charges reported to a national insurance database as a surrogate for costs here. This is a major point and warrants significant discussion. Charges are not a surrogate for costs, at least in the American system to which I am familiar. I highly doubt they are in Korea, either. The cost of a procedure or service indicates how much the provider must spend to provide that service, such as infrastructure, salaries, medications, and supplies. Readers of this manuscript are likely going to be unfamiliar with the Korean medical financing system, so the authors should briefly explain this. If the hospitals are private entities, what constraints do they have on their charges? How do they determine the charge for a procedure or visit? How closely do the authors believe the charges represent the true cost of providing a procedure or visit? If they are freely set by the private institutions and responsive to market pressures, they are likely a good approximation. If they are set by the government or some planning body, they are unlikely to actually represent costs. The manuscript would benefit greatly from the authors both giving a brief explanation of the Korean healthcare financing system and of discussing how this would affect the relationship of charges to costs for this study.

Answer: Thank you very much for raising this issue. Your concern is correct, and this is an important issue to be clarified in this paper. The ROK adopted a single-payer system (National Health Insurance System) to provide a fair opportunity to all people in the ROK. The cost of medical services and supplies is determined by the board in the NHIS, and the fee is set by the relative value of the service, which considers the cost of infrastructure, instruments, supplies and services by medical staff. All hospitals and doctors should follow the regulations for reimbursement by law and the cost is almost the same across all hospitals except for uninsured costs, such as using anti-adhesive agents, hemostatic agents and artificial bone material. The uninsured cost comprises approximately 35% of the direct medical cost. The proportion of uninsured costs is open to the public, and the proportion is controlled by periodic accounting audits by national tax services. However, as you noted, the charge here just reflect the utilization of NHIS and it underestimate cost for such as infrastructure and use of medical supplies, Therefore, the charge does not represent actual cost of hospital but a utilization of NHIS. We acknowledged this problem and tried to make a realistic cost system, but it was not easy with a limited budget of NHIS. The issue should be discussed with data obtained for that purpose. Thank you again for your concern and for giving us the opportunity to explain the NHIS of the ROK. A brief introduction of the NHIS and charge system has been added to the revised manuscript as follows.

Page 10, line 3

National Health Insurance System in Republic of Korea[14]

The ROK adopted a government-controlled NHIS, and it is funded by the taxpaying citizenries. The NHIS is a service provider, and the Health Insurance Review & Assessment Service (HIRA, https://www.hira.or.kr/eng/main.do) controls approvals of reimbursement. In Korea, the hospital type is defined by law [14]. General hospitals have at least 7 departments with more than 99 beds, including internal medicine, general surgery, obstetrics and gynecology, pediatrics, diagnostic radiology, anesthesiology, pathology, and laboratory medicine, with at least 1 board-certified doctor in each department [9]. Tertiary-referral hospitals are designated from among the general hospitals by the government. A tertiary-referral hospital should have at least 20 departments and should include the basic requirements of a general hospital along with having a residency training program, at least 5 operating rooms, and a variety of imaging/diagnostic tools used for computed tomography, magnetic resonance imaging, electromyography, angiography, gamma camera radiography, and Holter cardiac monitoring. In addition, the proportion of patients with difficult diseases (as designated by the Minister of Health and Welfare) should be more than 12% of the total number of annual inpatients [9, 14]. The final type, hospitals, is defined as a hospital lacking any of the essential departments or having between 30 and 99 beds. Private clinics have fewer than 30 beds [9]. The NHIS allows people free to choose medical service providers. The deduction rate is 50% for outpatient clinics and 20% for admission (https://hineca.kr/1913). A "fee for service” (FFS) is the traditional reimbursement system, and hospitals request reimbursement after outpatient clinics or discharge of patients. The fee is set by the relative value of the service, which considers the cost of infrastructure, instruments, supplies and services by medical staff [17, 18]. However, the fee may underestimate an actual cost of hospital to provide medical service. Therefore, the direct cost (charge to NHIS) may not represent actual cost of hospital but may represent a utilization of NHIS. The reimbursement was approved by the review board in HIRA. For reimbursement, the disease codes were standardized based on the international classification of diseases, 10^th^ version (ICD-10). The codes of medical services are standardized by the NHIC and HIRA to file claims for medical fees to the NHIC. All types of hospitals should follow the standardized codes of diseases and procedures for reimbursement by law. The total insured direct costs (which are covered by the NHIS during admission and clinical visits) are recorded in the HIRA database, but the uninsured direct costs, such as using anti-adhesive agents, hemostatic agents and artificial bone material, are not recorded and are known to comprise 35% of the direct medical cost [19].

Reviewer #2: This is an interesting study especially with long follow-up up to 10 years. Several issues that need to be addressed include the following:

Answer:

Thank you very much for your encouraging comments. I respect valuable comments by the reviewer, and the manuscript has been revised accordingly. Please see the changes throughout the manuscript.

#1. The cost of non-surgical management varies across countries and that contributes significantly to the data presentation.

Answer: I appreciate your comment. The cost of nonsurgical management varies, and the cost may not be assessable in many countries. The purpose of this study was to provide an overview of the utilization of the National Health Insurance System (NHIS) and was not intended to give a full overview of the cost. In addition to noninsured costs, indirect medical costs, such as job loss and quality of life, were not covered in this study. Therefore, this result should be interpreted as such, and the interpretation of this result may differ based on the different insurance systems and willingness to pay by society. Moreover, the interests of private insurance companies and hospitals should be considered. We clarified this in the revised manuscript as follows and underscored the message in the conclusion.

Page 20, line 24

To the best of our knowledge, the present study is the first to compare direct medical costs between surgery and nonsurgical treatment under NHIS over a long-term follow-up period [12, 13]. A direct medical costs for both Western and Oriental medicine are considered and these results may be helpful in countries with the similar medical system. However, the present study has several limitations. As mentioned, the claim data lack detailed clinical and radiological information, which limits the direct application of the results to clinical practice [5, 28, 29]. Common clinical indicators such as quality of life or disability index would be helpful to better compare surgery and nonsurgical management. Second, the current results cannot be generalized because the thresholds and indications of surgery and the outcomes of treatments are not uniform across doctors and countries, and the NHIS influences the indications by enforcing reimbursement restrictions [5, 47]. Moreover, claims data do not assess the over/underuse of treatments by physicians or over/underutilization of insurance services by patients. Third, the costs here do not account for uninsured costs or societal costs such as missed work or quality of life. [13, 48]. Medical fees for services that are not covered by insurance represent approximately 35% of medical costs in the ROK, and the actual direct costs can be estimated as the total cost*(100/65)[19]. Societal costs could not be assessed by a secondary data that lacks clinical information, employment and functional status. In addition, as with most cost-effectiveness studies, specific dollar values might not be equivalent among studies, and the WTP considering the benefits and risks of each surgical method in different countries may lead to different interpretations [35, 43]. Fourth, only age, sex, diagnosis, osteoporosis, diabetes and previous direct medical costs were considered for the matching process, and other important comorbidities, such as smoking, hypertension and congestive heart failure, and socioeconomic factors such as income and employment status, were not considered. Although the result seemed to provide a general overview of the direct medical costs, the current results posed a risk of bias due to potential incomplete matching. Regardless of these limitations, the present study provides an overview of how the NHIS is utilized for lumbar degenerative spinal disease on a nationwide scale, and this information may be helpful for physicians, patients and policy makers to improve the NHIS.

Page 22, line 3

The results of the present study should be interpreted in light of the costs of medical services, indirect costs, societal cost, quality of life and societal WTP in each country.

#2. The extent of patients identified as disabled who did not return to work needs to be analyzed because the indirect cost of a leave of absence can offset some of the early costs of surgery.

Answer: I appreciate your comment, and fast recovery is a very important issue after either surgery or nonsurgical treatment. As you mentioned, a fast recovery would offset some of the early surgical costs. Although the job status data were not in the NHIS database and could not be used to interpret the present study, we searched relevant papers and added the idea in the discussion section as follows.

Page 19, line 12

Therefore, according to the NHIS results, conservative treatments may be cost-effective in the ROK. However, the issues of societal cost and indirect cost were not addressed in this study. A fast recovery after surgery, reducing indirect medical costs by shortening the period of job loss, reducing direct medical costs by reducing the cost of nonsurgical management and improving quality of life would offset some of the early costs of surgery [20, 32, 33]. One study from the Sweden health data registry showed that indirect costs comprised 57% of the total cost per patient [34]. Therefore, reducing indirect costs by shortening sick leave from jobs and improving quality of life would effectively reduce total medical costs, and surgery may be a cost-effective option from a long-term economic perspective [32]. Various factors such as the cost of medical services, indirect medical costs and societal WTP vary according to the medical system and economy of each society should be considered together to interpret those results [6, 34-39].

#3. The reoperation rate was especially high in the surgical group, necessitating better clarification/stratification of this data for interpretation.

Answer: I appreciate this observation. Because I used the label ‘reoperation’, there may have been confusion. Failure was defined as reoperation in the surgery cohort and operation in the nonsurgical cohort. Therefore, failure of initial treatment may be an appropriate label. In addition, reoperation after surgery included reoperation at the index level and at the other lumbar levels. Therefore, surgery at the other lumbar levels was not reoperation. This explains the high reoperation rate in the surgical group We clarified this in the revised manuscript as follows.

Page 9, line 17

Treatment failure was defined as any kind of lumbar spinal surgery at any lumbar level being performed after the initiation of either surgical or nonsurgical treatment[8, 9, 11, 14]. Because the exact lumbar level was not recorded in the registry, treatment failure after surgery was included at both the index surgery (reoperation) and the other lumbar levels [8, 9, 11, 13, 14].

Table 5. Failure of treatment

| Cohort | Surgery | | | Nonsurgical | | | P-value |
| --- | --- | --- | --- | --- | --- | --- | --- |
|  | Patients | Failure* | Rate (%) | Patients | Failure | Rate (%) |  |
| Lumbar disc herniation | 1,229 | 195 | 15.87 | 1,229 | 86 | 7.00 | < 0.01 |
| Spinal stenosis without Spondylolisthesis | 1,059 | 171 | 16.15 | 1,059 | 106 | 10.01 | < 0.01 |
| Spinal stenosis with spondylolisthesis | 360 | 42 | 11.67 | 360 | 48 | 13.33 | > 0.05 |
| Spondylolysis | 50 | 2 | 4.00 | 50 | 5 | 10.00 | > 0.05 |
| total | 2,698 | 410 | 15.20 | 2,698 | 245 | 9.08 | < 0.01 |

*Failure after surgery included both surgery at the index level (reoperation) and the other lumbar levels,

#4. Figure 2 and tables are unclear and needs better organization and clarification.

Answer: I appreciate your observations and have revised Figure 2 and the tables to improve the organization and for clarification. Please see the revised fig. 2 and tables embedded in the revised manuscript.

Fig 2. Monthly cost after treatment

The average monthly costs and standard errors are plotted in the graph. Asterisk (*) implies statistically significant difference between surgery cohort and nonsurgical cohort.

#5. State the percentage of patients who had long-term follow-up. Determine the number of patients at each follow-up point.

Answer: I appreciate this comment. The cohort was constructed from patients between 2006 and 2008, and nearly all patients were followed up with encrypted identification codes until 2015. At 10 year, 92% of surgical cohort were followed-up and 90% of nonsurgical cohort were followed-up. We revised Figure 1 to show follow-up rates.

Page 8, line 7

The resident registration number was replaced with a newly assigned eight-digit personal ID, which enables longitudinal follow-ups to be performed for nearly all people until 2015 [12].

Fig 1. Flow diagram of patients

The number of patients were described in the box. The follow-up rates were marked along with time points. The detailed inclusion and exclusion criteria are described in the manuscript.

#7. The quality of life and disability index score such as ODI would be helpful to better compare both groups.

Answer: I truly appreciate your comment, and this is the most important issue by which clinical studies supersede studies of secondary data. I acknowledge the limitation of using secondary data lacking clinical information and noted it in the limitation section as follows.

Page 21, line 2

However, the present study has several limitations. As mentioned, the claim data lack detailed clinical and radiological information, which limits the direct application of the results to clinical practice [5, 28, 29]. Common clinical indicators such as quality of life or disability index would be helpful to better compare surgery and nonsurgical management. Second, the current results cannot be generalized because the thresholds and indications of surgery and the outcomes of treatments are not uniform across doctors and countries, and the NHIS influences the indications by enforcing reimbursement restrictions [5, 47]. Moreover, claims data do not assess the over/underuse of treatments by physicians or over/underutilization of insurance services by patients. Third, the costs here do not account for uninsured costs or societal costs such as missed work or quality of life. [13, 48]. Medical fees for services that are not covered by insurance represent approximately 35% of medical costs in the ROK, and the actual direct costs can be estimated as the total cost*(100/65)[19]. Societal costs could not be assessed by a secondary data that lacks clinical information, employment and functional status.
