# Supplementary material for: Direct medical costs after surgical or nonsurgical treatment for degenerative lumbar spinal disease: A nationwide matched cohort study with a 10-year follow-up
Source: PLoS One. 2021 Dec 1;16(12):e0260460. doi: 10.1371/journal.pone.0260460 (PMC8635587; doi:10.1371/journal.pone.0260460)
Supplement: Attachment — Submitted filename: Response to reviewers_20211026.docx [file pone.0260460.s003.docx]

Please include the following items when submitting your revised manuscript:

- A rebuttal letter that responds to each point raised by the academic editor and reviewer(s). You should upload this letter as a separate file labeled 'Response to Reviewers'.
- A marked-up copy of your manuscript that highlights changes made to the original version. You should upload this as a separate file labeled 'Revised Manuscript with Track Changes'.
- An unmarked version of your revised paper without tracked changes. You should upload this as a separate file labeled 'Manuscript'.

Reviewer #1: The authors have done an outstanding job addressing most of my previous concerns. My one lasting issue is with the discussion of “cost” versus “charges.” The authors have included an excellent overview of the Korean healthcare financing system. I applaud them for that. From the author’s explanation, the costs they report are based on a centrally set reimbursement fee by the Korean government. Thus, they don’t represent true costs. They state in their response to my comment that the “cost” of medical services and supplies is determined by the board of the NHIS. A more accurate description would be that the “reimbursement” is determined by the board of NHIS. This is similar to the United States. The government may set a reimbursement for a surgical procedure, but that reimbursement is not reflective of the true cost. The administrators who set that rate may try to approximate cost, but costs are ever shifting and determined by the market. If, for example, the cost of labor increases (as has happened recently), the costs of surgical procedures will increase. However, the reimbursement rate from the government will remain the same, regardless of how much the hospital must pay out for staffing, electricity, supplies, etc. True determination of cost requires a thorough accounting including time-driven activity-based costing, which is far outside the scope of this paper.

While these economic nuances need not be included in this particular manuscript, we should ensure that the conversation around surgical costs is appropriately phrased. For example, in the conclusion, instead of stating “surgery was initially more costly,” it would be more appropriate to say “surgical treatment initially led to more government reimbursement than nonsurgical…” or “cost the government payor more…” or something along those lines. The authors should ensure that the language in the paper does not suggest that the monetary figures are implied to be actual economic costs but instead reflect reimbursement charges from the government.

Answer.

I appreciate for the reviewer’s concern and valuable suggestion. I totally agree with your opinion that the “reimbursement” is determined by the board of NHIS. Hence, this paper has a limitation that true determination of cost requires a thorough accounting including time-driven activity-based costing, which is far outside the scope of this paper. Therefore, I clarified that the costs in this paper means a charge to government for a reimbursement, which does not reflect the actual economic cost in the revised manuscript including limitation section.

Abstract

Results

The characteristics and matching factors were well-balanced between the matched cohorts. Overall, surgery cohort spent $50.84/patient/month, while the nonsurgical cohort spent $29.34/patient/month (p<0.01). Initially, surgery treatment led to more charge to NHIS ($2,762) than nonsurgical treatment ($180.4) (p<0.01). Compared with the non-surgical cohort, the surgery cohort charged $33/month more for the first 3 months, charged less at 12 months, and charged approximately the same over the course of 10 years.

Conclusion

Surgical treatment initially led to more government reimbursement than nonsurgical treatment, but the charges during follow-up period were not different. The results of the present study should be interpreted in light of the costs of medical services, indirect costs, societal cost, quality of life and societal willingness to pay in each country. The monetary figures are implied to be actual economic costs but those in the reimbursement system instead reflect reimbursement charges from the government.

Page 9, line 8

During the last 3 years, the standard deviation of the direct medical cost (charge to NHIS) in 3,881 patients was $730, and a difference of no more than $73 was considered a similar severity of disease in the matching process.

Page 11, line 22

Therefore, the imbursement by NHIS does not represent actual cost of hospital but represents a utilization of NHIS.

Page 22, line 5

Lastly, but not least, this paper has a limitation that true determination of cost requires a thorough accounting including time-driven activity-based costing, which is far outside the scope of this paper. The present monetary figures were a charge to government for a reimbursement, which does not reflect the actual economic cost.

Conclusion

Surgical treatment initially led to more government reimbursement than nonsurgical treatment, but the charges during follow-up period were not different. The results of the present study should be interpreted in light of the costs of medical services, indirect costs, societal cost, quality of life and societal WTP in each country. The monetary figures are implied to be actual economic costs but those in the reimbursement system instead reflect reimbursement charges from the government.

Reviewer #2: (No Response)

Answer.

I appreciate your advices for the manuscript.
